# Postmarketing surveillance study of asciminib in patients with resistant/intolerant chronic myeloid leukemia in Japan

**DOI:** 10.1007/s12185-026-04199-x

**Published:** 2026-03-31

**Authors:** Kazuaki Yamaguchi, Makoto Aoki, Ryohei Osako

**Affiliations:** Clinical Development Japan CDD 3, Novartis Pharma K.K., Toranomon Hills Mori Tower, 23-1, Toranomon 1-Chome, Minato-ku, Tokyo, 105-6333 Japan

**Keywords:** Asciminib, CML, Real world, Japan, Postmarketing surveillance

## Abstract

Following asciminib’s initial approval in Japan for chronic myeloid leukemia (CML) resistant or intolerant to previous therapy, this all-patient, postmarketing surveillance study was initiated. Predefined safety specifications, overall safety, and effectiveness were assessed for 48 weeks from asciminib initiation (safety analysis set, n = 523). The approved daily dose of asciminib (80 mg [40 mg twice daily]) was most frequently used (63.9%) and not exceeded. Discontinuations (28.5%) were primarily due to adverse events (18.2%), including disease progression. Incidences of the safety specifications myelosuppression, pancreatitis, QT interval prolongation, infections, vascular occlusive events, and photosensitivity were 8.6%, 4.4%, 2.5%, 1.3%, 0.2%, and 0%, respectively, with low rates of treatment discontinuation for these events. There were no notable trends in the rates or types of adverse drug reactions in patients aged ≥ 65 years or with concurrent renal impairment, hepatic impairment, or cardiac dysfunction. The cumulative major molecular response rate was 61.2% by week 48 and was not affected by age (≥ 65 years) or comorbidities. Cumulative MR^4.0^ and MR^4.5^ rates by week 48 were 42.3% and 26.5%, respectively, with some patients harboring baseline *BCR::ABL1* mutations showing these responses. These real-world outcomes support the safety and effectiveness of asciminib for patients with resistant/intolerant CML.

**Supplementary Information:**

The online version contains supplementary material available at 10.1007/s12185-026-04199-x.

## Introduction

Chronic myeloid leukemia in chronic phase (CML-CP) is a myeloproliferative neoplasm driven by the activity of the constitutive BCR::ABL1 fusion oncoprotein [1]. First-generation and second-generation (1G and 2G) tyrosine kinase inhibitors (TKIs), such as imatinib, nilotinib, bosutinib, and dasatinib, are competitive inhibitors at the adenosine triphosphate (ATP)-binding site [2] and common treatments for patients with CML; however, these TKIs have varying safety profiles and the long-term use can cause complications [3–5]. In contrast, asciminib is the first BCR::ABL1 inhibitor intentionally designed to Specifically Target the ABL Myristoyl Pocket (STAMP) [6]. Given that asciminib binds to a separate region of the BCR::ABL1 protein to the ATP-competitive TKIs, off-target activities are limited, which may translate into improved safety and the potential to overcome resistance to the ATP-competitive TKIs [6, 7].

In the phase 3 ASCEMBL trial, the superior efficacy and the favorable safety and tolerability of asciminib compared with the 2G TKI bosutinib were demonstrated at week 24 in patients with CML-CP who had previously been treated with ≥ 2 TKIs [8]. At week 24, major molecular response (MMR) was improved with asciminib versus bosutinib (25.5% vs. 13.2%, respectively) and fewer grade ≥ 3 adverse events (AEs) and AEs leading to discontinuation were observed for asciminib (50.6% vs. 60.5% and 5.8% vs. 21.1%, respectively) [8]. A subsequent analysis showed that the efficacy and safety outcomes observed for asciminib in Japanese patients from ASCEMBL were consistent with those in the overall study population [9]. Notably, these benefits were sustained in both the overall and Japanese populations at the 96-week analysis of ASCEMBL [10, 11], and after 4 years of follow-up the superiority of asciminib over bosutinib was still evident [12]. Based on the outcomes from the ASCEMBL study [8], asciminib was approved in the US and Europe for patients with CML-CP who had previously been treated with ≥ 2 TKIs [13, 14]. It was also approved in the US for patients with a T315I mutation, based on outcomes from a phase 1, first-in-human study [15]. In 2022, asciminib (40 mg twice daily [bid]) was approved in Japan for patients with CML who were resistant or intolerant to prior TKIs. Most recently, asciminib has demonstrated superior efficacy and a favorable safety profile versus all currently available TKIs in patients with newly diagnosed CML in the ASC4FIRST study [16] and is now indicated for the treatment of patients with newly diagnosed CML in addition to previously treated CML in the US [14]. As with ASCEMBL, the outcomes in Japanese patients from ASC4FIRST reflected those of the overall study population [17] and in 2025, asciminib (80 mg once daily [qd]) was approved in Japan for patients with CML at any treatment stage [18, 19]. Currently, asciminib is recommended as a third-line or later treatment option for patients who are resistant or intolerant to prior TKIs in the Japanese Society of Hematology (JSH) practical guidelines [5], which reflects the initial indication of asciminib following its approval in Japan in 2022.

CML treatment requires long-term administration, and therefore, the chronic issues faced by patients are a key concern, including quality of life (QOL) and tolerability [3, 20, 21]. Patients requiring third-line or later-line therapy for CML often face multiple challenges, including a decreasing likelihood of response, development of resistance, and long-term safety and intolerance issues [22]. The JSH practical guidelines [5] highlight the different safety profiles of the TKIs and note that patient characteristics and comorbidities need consideration when selecting a TKI, including the identification and monitoring of patients who may be at risk of specific AEs (such as cardiovascular AEs) with the long-term use of TKIs. Likewise, the European LeukemiaNet (ELN) recommendations highlight the need to take into account comorbidities, minimize side effects and improve QOL as key elements of long-term CML management; this includes consideration of patient comorbidities and circumstances when choosing or switching TKIs [20]. One of the most common reasons for discontinuing or switching treatment with ATP-competitive TKIs is intolerance [23, 24], which may result in poor adherence that can adversely affect patient response to treatment [25–27].

Following its approval for patients with resistant or intolerant CML in Japan, a postmarketing surveillance (PMS) study was initiated to primarily assess the safety of asciminib in a real-world setting in Japan.

## Methods

### Ethics

The study protocol was reviewed by the Pharmaceuticals and Medical Devices Agency (PMDA) in Japan. Informed consent from patients was not a requirement for this study as per Good Post-marketing Study Practice (GPSP); however, data from any sites and/or patients who did not provide consent for the publication of their data were not included in this publication.

### Patients and study design

This was an uncontrolled, central registration system, all-case, multicenter, observational, special drug-use surveillance study conducted in accordance with GPSP ordinance in Japan. All patients who started asciminib treatment from the date of its approval in Japan (March 28, 2022) to February 28, 2023, were eligible for inclusion in the study.

Patients needed to be registered for the study within 30 days of starting asciminib treatment, or within 30 days of the study site initiating the study for those patients who were already receiving asciminib in routine clinical practice. Investigators treated patients according to the asciminib label in Japan at the time of the study.

**Fig. 1:**
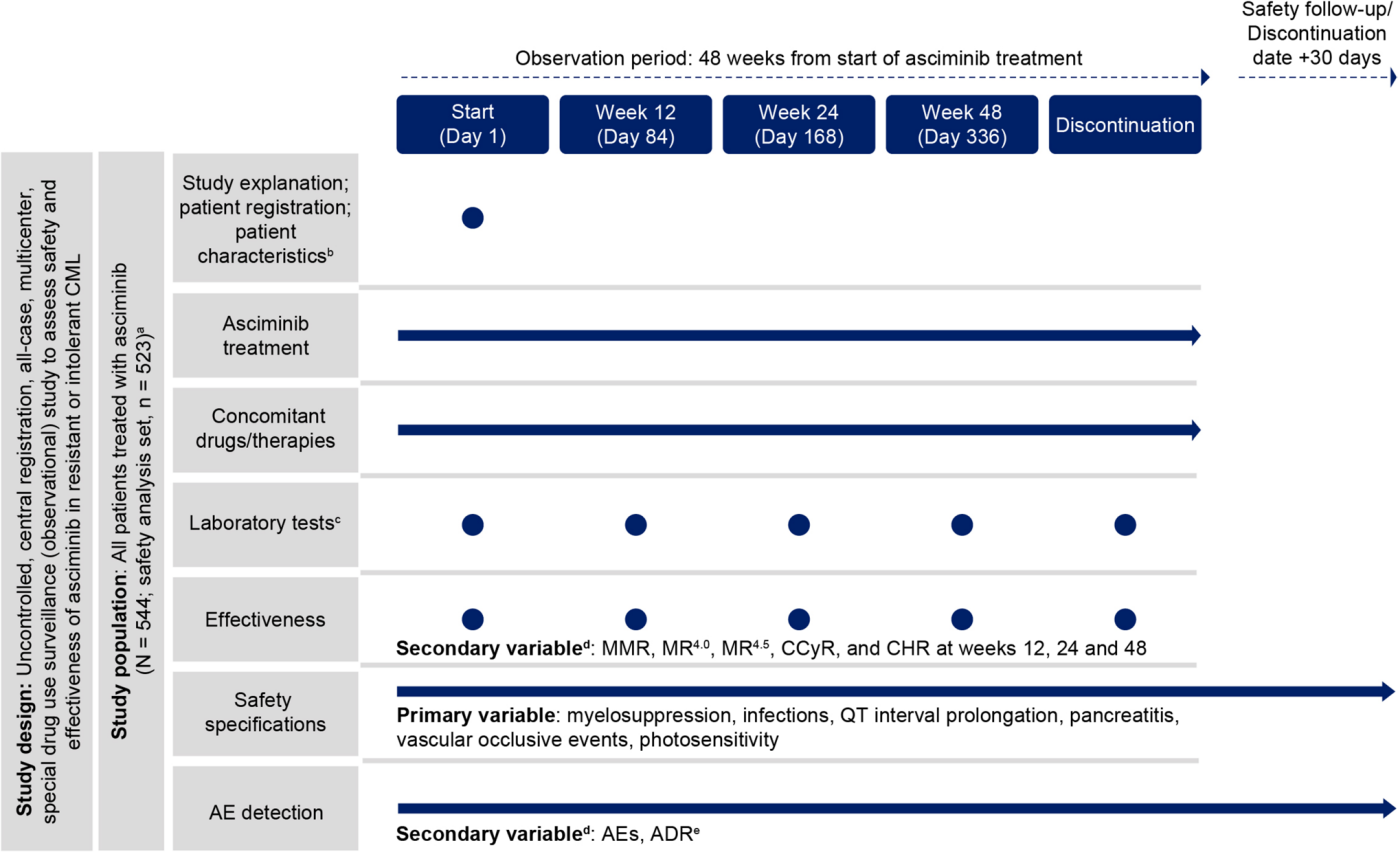
Study design and assessment schedule for asciminib in patients with resistant/intolerant CML. *ADR* adverse drug reaction, *AE* adverse event, *CCyR* complete cytogenetic response, *CHR* complete hematological response, *CML* chronic myeloid leukemia, *ECG* electrocardiogram, *MMR* major molecular response, *MR* molecular response, *WBC* white blood cell; ^a^Registration period was July 2022–November 2023, end of study was July 2024 (current analysis cut-off: July 4, 2024); ^b^Included sex, age, reasons for asciminib use, date of diagnosis, medical history, concurrent conditions, previous drugs for underlying disease, presence/absence of resistance/intolerance to previous drugs and details, treatment line number, *BCR::ABL1* mutations; ^c^Vital signs (blood pressure, ECG [QTc interval]), hematology (WBC count, differential WBC count [neutrophil, lymphocyte, basophil, eosinophil, monocyte], platelet count) and blood chemistry (amylase, lipase); ^d^Key endpoints listed only (safety and effectiveness also assessed by patient characteristic and treatment line); ^e^ADR defined as an AE suspected by investigator to be causally related to treatment, or causality is not recorded

Patients in this PMS were assessed for 48 weeks from the start of asciminib treatment (Fig. 1). Any patients who discontinued treatment during this observation window were followed for 30 days post last asciminib administration to assess safety. The study was conducted from May 13, 2022, to July 4, 2024. Patients were registered between July 4, 2022, and November 28, 2023.

### Study objectives and endpoints

The aims of this study were to investigate the occurrence, severity, and clinical course of the safety specifications of asciminib, to identify factors involved in their occurrence, and to assess the clinical safety of asciminib in patients with resistant/intolerant CML.

The primary objective was to assess the safety profile of asciminib with respect to the safety specifications (myelosuppression, infections, QT interval prolongation, pancreatitis, vascular occlusive events [important identified risks], and photosensitivity [important potential risk]), as defined in the Japanese Risk Management Plan (J-RMP) (Supplementary Table 1). The secondary objectives included assessment of the overall safety profile and effectiveness of asciminib, including by patient characteristic, comorbidity, and treatment line. Safety was assessed by type, frequency, and severity of adverse drug reactions (ADRs) and AEs, including the safety specifications. For effectiveness, major molecular response (MMR, *BCR::ABL1*^*IS*^ ≤ 0.1%), MR^4.0^ (*BCR::ABL1*^*IS*^ ≤ 0.01%), MR^4.5^ (*BCR::ABL1*^*IS*^ ≤ 0.0032%), complete cytogenetic response (CCyR), and complete hematological response (CHR) were assessed. Rates of patients with *BCR::ABL1* mutations were also captured.

### Assessments

Data were collected electronically (by electronic data capture [EDC]) or manually (case report forms [CRFs]) as part of routine medical practice, which determined the visit frequency and assessment variables.

Patient characteristics, laboratory test outcomes, and gene mutation status were collected at/before asciminib treatment initiation, while baseline treatment response was assessed within 30 days before the start date of treatment. During the observation period, treatment response was assessed at weeks 12, 24, and 48, and at discontinuation, and details captured on asciminib treatment, discontinuations and interruptions, concomitant medications and therapies, laboratory tests, and the presence of *BCR::ABL1* mutations, with safety assessed throughout (Fig. 1; Supplementary information).

Treatment response was assessed by the investigator according to ELN 2020 recommendations [3] and JSH Practical Guidelines for Hematological Malignancies [28]. Concomitant/previous drugs were coded using the Iyakuhinmei Data File and concurrent conditions and AEs coded using the Japanese version of the Medical Dictionary for Regulatory Activities (MedDRA/J; version 27.0) and by Common Terminology Criteria for Adverse Event (CTCAE).

### Statistical analysis

The sample size for this PMS was based on incidence rates for the safety specifications in the ASCEMBL study. Assuming that similar rates would be observed in this PMS, the sample size was 400 for the assessment of the safety specifications defined in the primary endpoint (Supplementary information). The sample size needed to maintain the width of the 95% confidence intervals (CIs) at ≤ 10% was calculated for each safety specification. To achieve the enrollment of 400 patients as the safety analysis set, 440 patients needed to be registered, assuming a 10% drop out rate. Safety analysis set included all CRF-locked patients who did not meet any safety analysis exclusion criteria (Supplementary information).

Patients who continued treatment for the 48 weeks (336 days) were considered as completing the observation period; patients were considered as discontinued if they stopped treatment before 48 weeks (336 days). All analyses were performed using SAS version 9.4.

For qualitative data, frequency counts and percentages were provided. Descriptive statistics were provided for quantitative data.

The safety analysis set was used for all safety analyses. ADRs were defined as AEs suspected to be causally related to asciminib or where the causality was not recorded. Incidence rates and 95% CIs are provided for ADRs of the safety specifications. The Clopper–Pearson method was used for the estimation of the 95% CI. Odds ratios (ORs) and 95% CIs for ADRs by demographics and patient characteristics were estimated as univariate analyses, and a logistic regression model was used to estimate the adjusted ORs and 95% CIs of ADR in the multivariate analysis. Those factors that did not include 1 as part of the 95% CI of ORs estimated by univariate analysis and predefined factors considered to be clinical meaningful variables (treatment line number of asciminib and presence/absence of resistance/intolerance to previous drugs) were included in the logistic model of the multivariate analysis.

For MMR, MR^4.0^ and MR^4.5^, CCyR and CHR rates, the molecular, cytogenetic, and hematological response sets, respectively, were used. Response rates and 95% CI were estimated at/by each timepoint and patients with missing/not recorded assessments were included in the denominator for estimating the rates. The Clopper–Pearson method was used for the estimation of 95% CI. Further details of the response analysis sets are provided in the Supplementary information.

## Results

### Patient disposition and baseline characteristics

**Fig. 2:**
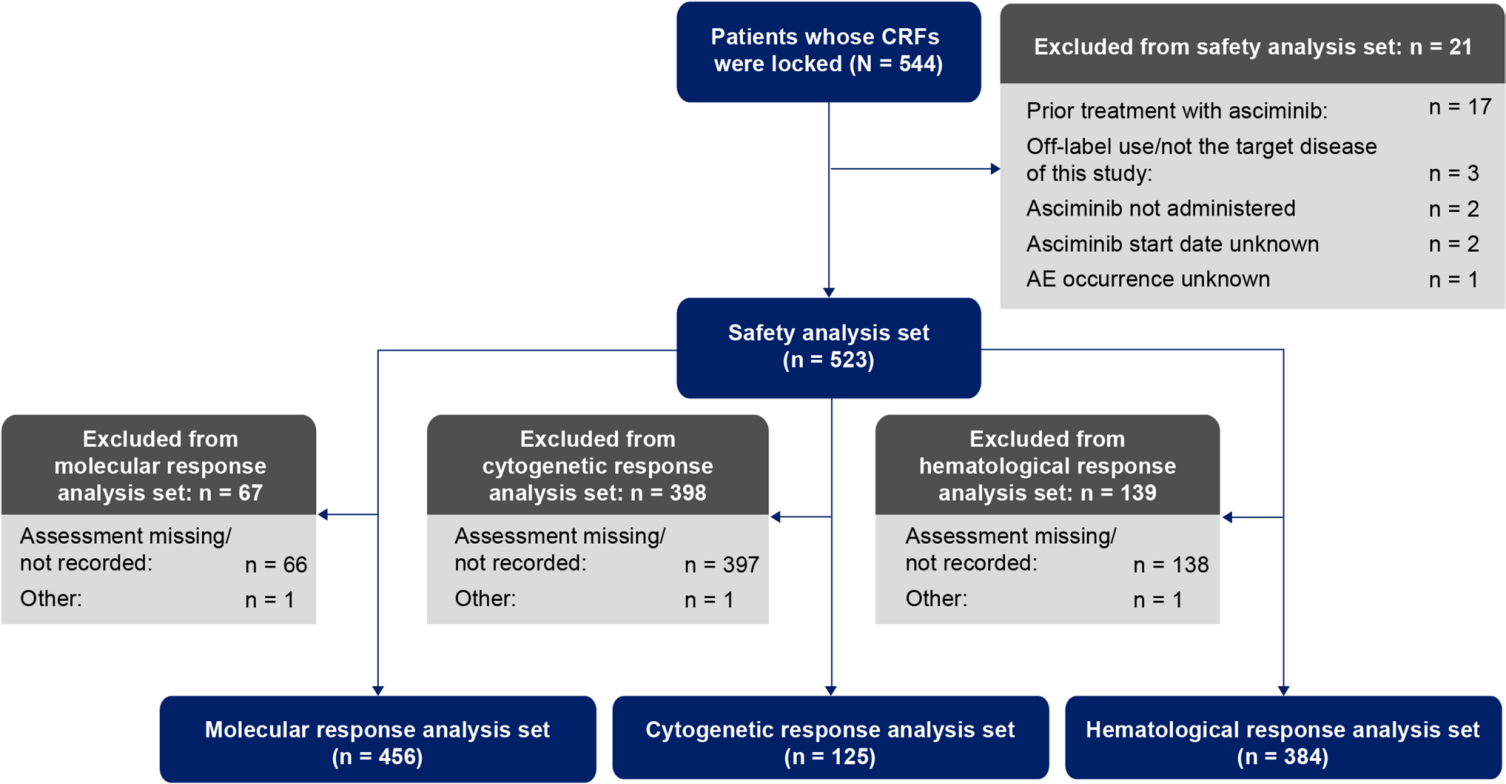
Patient disposition and analysis sets. *AE* adverse event, *CRF* case report form. Patients who had multiple reasons for exclusion from the safety analysis set, molecular response analysis set, cytogenetic response analysis set, or hematologic response analysis set were counted under each respective reason for exclusion, as such the number of patients included across these reasons may exceed the number of patients excluded

In total, data were available from 544 patients, of which 523 patients were included in the safety analysis set and 456 patients in the molecular response analysis set (Fig. 2). Patients had a median age of 69 years (range 14–92 years) and a median duration of CML of 3.47 years (range 0.0–30.5 years). In the safety analysis set, 71.3% of patients had underlying conditions, including cardiac dysfunction (20.5%). Approximately, one-third of patients (n = 158) were assessed for *BCR::ABL1* mutations at baseline, 25 (4.8%) of these were positive with mutations that included, for example, T315I and V299L (Table 1). Patients were heavily pretreated, 96.9% were receiving asciminib as a third-line or later-line treatment (Table 1). The most common previous treatments were 2G TKIs (dasatinib [81.5%], bosutinib [62.9%], and nilotinib [53.2%]; Supplementary Table 2) with intolerance being the most frequent reason for switching TKI treatment (56.1–78.7% of patients versus 19.8–41.3% of patients for resistance, across treatments).

**Table 1:** Patient demographics and baseline characteristics (safety analysis set)

Factors	Safety analysis set n = 523
Sex, n (%)	
Male	316 (60.4)
Female	207 (39.6)
Age, years	
Mean (standard deviation)	64.4 (16.2)
Median (Min–Max)	69.0 (14–92)
Age, n (%)	
< 65 years	216 (41.3)
≥ 65 years	307 (58.7)
Duration of CML, years^a^	
Mean (standard deviation)	5.4 (5.4)
Median (Min–Max)	3.5 (0.0–30.5)^b^
Duration of CML, n (%)	
< 1 year	74 (14.1)
≥ 1 year to < 5 years	252 (48.2)
≥ 5 years	192 (36.7)
Unknown/not recorded	5 (1.0)
Concurrent condition, n (%)	
None	150 (28.7)
Yes	373 (71.3)
Concurrent condition: renal impairment, n (%)	
None	464 (88.7)
Yes	59 (11.3)
Concurrent condition: hepatic impairment, n (%)	
None	504 (96.4)
Yes	19 (3.6)
Concurrent condition: cardiac dysfunction, n (%)	
None	416 (79.5)
Yes	107 (20.5)
Treatment line number for asciminib, n (%)	
1	0
2	16 (3.1)
≥ 3	507 (96.9)
3	217 (41.5)
4	143 (27.3)
≥ 5	147 (28.1)
Type of previous treatment, n (%)	
1G TKI	2 (0.4)
2G TKI	149 (28.5)
3G TKI	1 (0.2)
1G + 2G TKI	183 (35.0)
2G + 3G TKI	117 (22.4)
1G + 3G TKI	2 (0.4)
1G + 2G + 3G TKI	69 (13.2)
*BCR::ABL1* mutations, n (%)	
Not assessed	365 (69.8)
Negative	133 (25.4)
Positive	25 (4.8)^c^

*CML* chronic myeloid leukemia, *G* generation, *TKI* tyrosine kinase inhibitor

^a^n = 518

^b^Duration of CML was calculated as 0.0 years in 3 patients (1 patient’s duration was 16 days and exact date of diagnosis was unknown in 2 patients)

^c^Mutations reported in these 25 patients were T315I, E255K, F317L, F359V, V299L, E255V, G250E, c550_822del, pL184_K274del, Pr362FS*21, c1086_1270del, R307W, and I418V. Safety analysis set (n = 523) used as the denominator to calculate the proportions (%). For the type of previous treatment, if the same type TKI was administered more than once, it was grouped into one type

### Asciminib treatment

Patients received asciminib for a median (range) of 336.0 days (1–336 days); 71.5% of patients received ≥ 48 weeks of asciminib treatment (Supplementary Table 3). Over half of patients (57.4%) initiated asciminib at the approved daily dose of 80 mg (40 mg bid), which remained the most frequent daily dose used during the study (63.9%) (Supplementary Table 3). Notably, no patients exceeded the approved dose (median [range] for the mean daily dose: 79.2 mg [2–80 mg]). The median (range) dose intensity was 76.9 mg/day (2–80 mg/day) and 46.5% of patients had a dose intensity of 80 mg/day, while 22.8% had a dose intensity of > 40 to < 80 mg/day (Supplementary Table 3).

Overall, of the 523 patients included in the safety analysis set, 149 (28.5%) discontinued treatment, primarily due to AEs (18.2%, including CML [disease progression]) and physician’s decision (5.2%). Other reasons provided were patient/family decision (3.6%) and missed visits (1.1%), one patient entered treatment-free remission (TFR, 0.2%), and for another the reason was not provided (0.2%).

### Safety

#### Adverse events

The incidence rates of AEs and grade ≥ 3 AEs in the safety analysis set (n = 523) were 46.8% and 18.9%, respectively, and 14.1% and 11.1% for SAEs and grade ≥ 3 SAEs. The most common AEs (reported in ≥ 5%) were platelet count decreased (5.7%) and CML (disease progression; 5.0%). Similarly, the most frequently reported SAE (reported in ≥ 1%) was CML (disease progression; 2.7%), then pneumonia and platelet count decreased (1.3% each), and CML transformation and neutropenia (1.0% each).

#### Adverse drug reactions

For ADRs, the incidence rate was 33.1% for all grade events and 11.7% for grade ≥ 3 events. The most common ADR and grade ≥ 3 ADR (≥ 1% of the safety analysis set) was platelet count decreased (5.2%; grade ≥ 3, 4.0%), other ADRs included rash, neutropenia, and electrocardiogram QT prolonged (Table 2). Based on the time of the first occurrence, most ADRs were reported early in the study, usually within the first 12 weeks of treatment (128 of 173 patients), thereafter 26 patients had ADRs during weeks 12–24 and 14 patients after week 24.

**Table 2:** Incidence of all grade and grade ≥ 3 ADRs (≥ 1%) in the safety analysis set

ADR (PT)	Safety analysis set n = 523	
	All grade n (%)	Grade ≥ 3 n (%)
Total	173 (33.1)	62 (11.7)
Platelet count decreased	27 (5.2)	21 (4.0)
Rash	15 (2.9)	0
Neutropenia	14 (2.7)	12 (2.3)
Electrocardiogram QT prolonged	13 (2.5)	7 (1.3)
Chronic myeloid leukemia^a^	9 (1.7)	3 (0.6)
Malaise	9 (1.7)	0
Diarrhea	8 (1.5)	0
Thrombocytopenia	7 (1.3)	5 (1.0)
Hypertension	6 (1.1)	2 (0.4)
Pleural effusion	6 (1.1)	1 (0.2)
Headache	6 (1.1)	0

When multiple AEs classified as PTs occurred in the same patient, the patient was counted once corresponding to the PT with the earliest date of occurrence. When the same AE classified as a PT occurred multiple times in the same patient, the patient was counted once corresponding to the first occurrence. Number of patients for the safety analysis set (n = 523) was used as the denominator to calculate the proportions (%). MedDRA/J version 27.0

*ADR* adverse drug reaction, *AE* adverse event, *MedDRA/J* Japanese version of Medical Dictionary for Regulatory Activities, *PT* preferred term

^a^Indicates disease progression

Serious ADRs (5.7%; grade ≥ 3 events, 4.2%) were most often platelet count decreased (1.0%), neutropenia (0.8%), and pneumonia (0.6%). Among ADRs leading to discontinuation (9.9%; grade ≥ 3 events, 3.4%), CML (disease progression) was the most common event (1.3%) (Table 3), whereas platelet count decreased and neutropenia (1.7% and 1.1% of patients, respectively) were the most frequent ADRs that resulted in treatment interruption (6.7%; grade ≥ 3, 4.2%) (Supplementary Table 4).

**Table 3:** Incidence of all grade and grade ≥ 3 ADRs leading to discontinuation in the safety analysis set

ADR (PT)	Safety analysis set n = 523	
	All grade	Grade ≥ 3
	n (%)	n (%)
Total	52 (9.9)	18 (3.4)
Chronic myeloid leukemia^a^	7 (1.3)	2 (0.4)
Platelet count decreased	5 (1.0)	5 (1.0)
Electrocardiogram QT prolonged	5 (1.0)	4 (0.8)
Myalgia	3 (0.6)	1 (0.2)
Nausea	3 (0.6)	1 (0.2)
Pancreatitis	3 (0.6)	1 (0.2)
Thrombocytopenia	2 (0.4)	1 (0.2)
Diarrhea	2 (0.4)	0
Pleural effusion	2 (0.4)	0
Rash	2 (0.4)	0
Vomiting	2 (0.4)	0
Anemia	1 (0.2)	1 (0.2)
Aspartate aminotransferase increased	1 (0.2)	1 (0.2)
Cardiac failure	1 (0.2)	1 (0.2)
Hypertension	1 (0.2)	1 (0.2)
Neutropenia	1 (0.2)	1 (0.2)
Abdominal distension	1 (0.2)	0
Alanine aminotransferase increased	1 (0.2)	0
Amylase increased	1 (0.2)	0
Blood pressure increased	1 (0.2)	0
Cytopenia	1 (0.2)	0
Decreased appetite	1 (0.2)	0
Dermatitis	1 (0.2)	0
Dysphagia	1 (0.2)	0
Eczema	1 (0.2)	0
Edema	1 (0.2)	0
Feces discolored	1 (0.2)	0
Headache	1 (0.2)	0
Heavy menstrual bleeding	1 (0.2)	0
Lipase increased	1 (0.2)	0
Neuropathy peripheral	1 (0.2)	0
Pain in extremity	1 (0.2)	0
Pruritus	1 (0.2)	0
Pulmonary hypertension	1 (0.2)	0
Renal disorder	1 (0.2)	0
Renal impairment	1 (0.2)	0
Toothache	1 (0.2)	0

ADRs are listed in descending order of incidence rate (based on all grade ADRs), and then in alphabetical order for ADRs of the same incidence rate. When multiple AEs are classified as PTs within the same system organ class for the same patient, the patient was counted once. When the same AE (PT) occurred multiple times in the same patient, the patient was counted once. Number of patients for the safety analysis set (n = 523) was used as the denominator to calculate the proportions (%). MedDRA/J version 27.0

*ADR* adverse drug reaction, *AE* adverse event, *MedDRA/J* Japanese version of Medical Dictionary for Regulatory Activities, *PT* preferred term

^a^Indicates disease progression

Three patients died due to ADRs (6 ADRs in total). One patient had 4 ADRs (decreased appetite, abdominal distension, nausea, and vomiting) that occurred 6 days into treatment; all were causally related to asciminib but the patient also had an underlying condition (cardiac failure) that was a suspected factor other than asciminib. The second patient had a worsening of CML recorded 24 days into treatment that was causally related to asciminib. In the third patient, the ADR (death) occurred 222 days after the start of treatment and was considered causally related to asciminib, but no further information was provided by the investigator.

### Safety specifications

In the safety analysis set (n = 523), incidence rates for AEs included in the 6 safety specifications were 10.1% for myelosuppression, 4.4% for infections, 2.9% for QT interval prolongation, 5.5% for pancreatitis, and 0.8% for vascular occlusive events. Similarly, ADRs occurred in 8.6%, 1.3%, 2.5%, 4.4%, and 0.2% of patients for myelosuppression, infections, QT interval prolonged, pancreatitis, and vascular occlusive events, respectively. There were no AEs or ADRs reported for photosensitivity. The first occurrences of ADRs for these safety specifications were usually early in treatment and did not tend to be prolonged in duration (Table 4). In addition, most cases of serious ADRs were resolved or resolving by the end of the safety follow-up period. Treatment discontinuations due to ADR were reported for myelosuppression (1.7%), pancreatitis (1.5%), and QT interval prolongation (1.0%), but were not observed for infection or vascular occlusive event. Full details of the ADRs, serious ADRs, and ADRs leading to treatment discontinuation and interruption for the safety specifications are provided in Supplementary Table 5.

**Table 4:** Incidence of safety specifications (first occurrence) in the safety analysis set

Safety specifications	Safety analysis set n = 523		Number of days to the first occurrence (days)^a^		Duration (days)^c^	
	n (%)	(95% CI)	n′^b^	Median (Min–Max)	m (%)^d^	Median (Min–Max)
Myelosuppression	45 (8.6)	(6.3, 11.3)	44	36.0 (2–253)	33 (73.3)	42.0 (5–379)
Infections	7 (1.3)	(0.5, 2.7)	7	104.0 (51–266)	7 (100)	17.0 (6–162)
QT interval prolongation	13 (2.5)	(1.3, 4.2)	13	23.0 (4–175)	12 (92.3)	35.5 (8–328)
Pancreatitis	23 (4.4)	(2.8, 6.5)	23	20.0 (1–313)	21 (91.3)	22.0 (1–336)
Vascular occlusive events	1 (0.2)	(0.0, 1.1)	1	2.0 (2–2)	0	–
Photosensitivity	0	(0.0, 0.7)	–	–	–	–

Number of patients in the safety analysis set (n = 523) was used as the denominator to calculate incidence rates. The Clopper–Pearson method was used to calculate 95% CIs. When multiple AEs classified as PTs within the same specification item occurred on the same day in the same patient, the number of days of the first event that was resolved or started resolving was adopted for duration. When there were incomplete dates and complete dates, complete dates were adopted

*AE* adverse event, *CI* confidence interval, *PT* preferred term

^a^The number of days to the first occurrence was: date of occurrence—start date of asciminib treatment + 1

^b^n′ represents patients for whom the number of days to the first occurrence was calculable. When multiple AEs classified as PTs within the same safety specification item occurred in the same patient, the number of days to the first occurrence of the first event was adopted for the number of days to the first occurrence. When there were incomplete dates and complete dates, complete dates were adopted

^c^Date of outcome when the first occurrence event was resolved or started resolving—date of the first occurrence + 1

^d^m represents the patients for whom the number of days to the resolution or improvement was calculable and m (%) is the number and proportion of patients whose outcome of the first occurrence event was “resolved” or “resolving”. The number of patients with each event (n) was used as the denominator to calculate the proportion (%)

### Potential risk factors and patients with special characteristics

The potential demographics and patient characteristics suspected to be related to the incidence of ADRs were investigated (Table 5). The factor that had the greatest influence on ADR incidence was age (≥ 65 years), with incidence rates of 36.5% and 28.2% in patients aged ≥ 65 years and < 65 years, respectively (OR 1.46, 95% CI 1.00, 2.13). However, when “treatment line number of asciminib” and “presence/absence of resistance/intolerance to previous drugs” predefined as the clinical meaningful variables were included in the multivariate logistic model with age, the adjusted OR for age was 1.35 (0.92, 1.99) and no significant difference was observed.

**Table 5:** ADR incidence analysis by demographics and patient characteristics

Factors	m	n (%)	Odds ratio (95% CI)
Safety analysis set	523	173 (33.1)	–
Sex			
Male	316	97 (30.7)	Reference
Female	207	76 (36.7)	1.31 (0.90, 1.90)
Age (children/Japan)			
< 15 years	1	0	< 0.01 (< 0.01, > 999.99)
≥ 15 years	522	173 (33.1)	Reference
Age (elderly)			
< 65 years	216	61 (28.2)	Reference
≥ 65 years	307	112 (36.5)	1.46 (1.00, 2.13)^a^
Age (late-stage elderly)			
< 75 years	361	111 (30.7)	Reference
≥ 75 years	162	62 (38.3)	1.40 (0.95, 2.06)
Duration of CML			
< 1 year	74	31 (41.9)	Reference
≥ 1 year to < 5 years	252	80 (31.7)	0.65 (0.38, 1.10)
≥ 5 years	192	61 (31.8)	0.65 (0.37, 1.12)
Unknown/not recorded^b^	5	1 (20.0)	–
Concurrent condition			
None	150	44 (29.3)	Reference
Yes	373	129 (34.6)	1.27 (0.84, 1.92)
Concurrent condition: renal impairment			
None	464	151 (32.5)	Reference
Yes	59	22 (37.3)	1.23 (0.70, 2.16)
Concurrent condition: hepatic impairment			
None	504	166 (32.9)	Reference
Yes	19	7 (36.8)	1.19 (0.46, 3.07)
Concurrent condition: cardiac dysfunction			
None	416	136 (32.7)	Reference
Yes	107	37 (34.6)	1.09 (0.70, 1.70)
Medical history^c^			
None	282	93 (33.0)	Reference
Yes	241	80 (33.2)	1.01 (0.70, 1.46)
Treatment line number of asciminib			
1	0	–	– (NE, NE)
2	16	3 (18.8)	0.46 (0.13, 1.63)
≥ 3	507	170 (33.5)	Reference
Type of previous treatment			
1G TKI	2	0	< 0.01 (< 0.01, > 999.99)
2G TKI	149	45 (30.2)	0.70 (0.44, 1.11)
3G TKI	1	0	< 0.01 (< 0.01, > 999.99)
1G + 2G TKI	183	70 (38.3)	Reference
2G + 3G TKI	117	34 (29.1)	0.66 (0.40, 1.09)
1G + 3G TKI	2	2 (100)	> 999.99 (< 0.01, > 999.99)
1G + 2G + 3G TKI	69	22 (31.9)	0.76 (0.42, 1.36)
Presence/absence of resistance/intolerance to previous drugs			
Resistance	75	19 (25.3)	Reference
Intolerance	276	101 (36.6)	1.70 (0.96, 3.02)
Resistance and intolerance	172	53 (30.8)	1.31 (0.71, 2.42)
Others^b^	0	–	–
*BCR::ABL1* mutations			
Not assessed	365	112 (30.7)	0.69 (0.46, 1.04)
Negative	133	52 (39.1)	Reference
Positive	25	9 (36.0)	0.88 (0.36, 2.13)

m represents the number of patients in the applicable category and n is the number of patients with ADRs. The denominator for the proportion (%) is m. The Wald's method was used to calculate the 95% CIs

*ADR* adverse drug reaction, *CI* confidence interval, *CML* chronic myeloid leukemia, *G* generation, *NE* not estimable, *TKI* tyrosine kinase inhibitor

^a^95% CI of the odds ratio did not include 1, suggesting that it may contribute to the high incidence rate of ADRs. The lower limit of the 95% CI is 1.00 due to rounding to the nearest whole number, the calculated value was slightly higher than 1

^b^“Unknown/not recorded” and “Others” were excluded from the calculation of odds ratio

^c^Significant medical history that had resolved prior to the start of asciminib treatment

No notable risk factors were observed in incidence rates or types of ADRs in patients aged ≥ 65 years, patients with concurrent renal impairment, hepatic impairment, or cardiac dysfunction (Table 5). Over half of the safety analysis set was aged ≥ 65 years (58.7%) and the overall incidence rate of ADRs was higher in patients ≥ 65 years than those < 65 years (Table 5); platelet count decreased was the most common ADR in both age groups (5.5% and 4.6%, respectively). Incidence rates were similar for patients with and without concurrent renal impairment (OR 1.23, 95% CI 0.70, 2.16), concurrent hepatic impairment (OR 1.19, 95% CI 0.46, 3.07), and concurrent cardiac dysfunction (OR 1.09, 95% CI 0.70, 1.70) (Table 5). Platelet count decreased (5.1%), and cardiac failure, renal impairment, electrocardiogram QT prolonged, and pancreatic enzymes (3.4% each) were the most common ADRs in patients with renal impairment. Rash, platelet count decreased, aspartate aminotransferase increased, and alanine aminotransferase increased (10.5% each) were the most frequently observed ADRs in patients with hepatic impairment. Electrocardiogram QT prolonged and platelet count decreased were the most common ADRs in patients with cardiac dysfunction (4.7% and 3.7%, respectively).

### ADR incidence by treatment line

For patients who received asciminib as second-line treatment (n = 16), ADRs occurred in 3 patients (18.8%); 1 patient (6.3%) had a grade ≥ 3 event (electrocardiogram QT prolonged) that resulted in discontinuation, but no serious ADRs or ADRs leading to interruption were reported. As expected, most patients received asciminib as their third-line or later-line treatment (n = 507) and in these patients, ADRs occurred in 33.5% (grade ≥ 3, 11.8%), serious ADR in 5.9%, and ADRs leading to treatment discontinuation or interruption in 10.1% and 6.9%, respectively (Supplementary Table 6). The most common ADRs were platelet count decreased (5.3%), rash (3.0%), and neutropenia (2.8%). Platelet count decreased (1.0%) and neutropenia (0.8%) were also the most frequent serious ADRs, whereas CML (disease progression; 1.4%) and platelet count decreased (1.8%) were the most common ADRs leading to treatment discontinuation and interruption, respectively. In patients receiving asciminib as the third line, fourth line, or ≥ fifth line of treatment, platelet count decreased was the most common ADR (4.1%, 4.2%, and 8.2%, respectively), with rash (4.1%) and electrocardiogram QT prolonged (4.2%) also common ADRs during third-line and fourth-line use. ADRs that most often resulted in discontinuation were CML (disease progression) in the third-line and ≥ fifth-line of treatment (1.4% and 2.0%, respectively) and platelet count decreased (2.1%) during the fourth-line treatment.

### Effectiveness

#### MMR

**Fig. 3:**
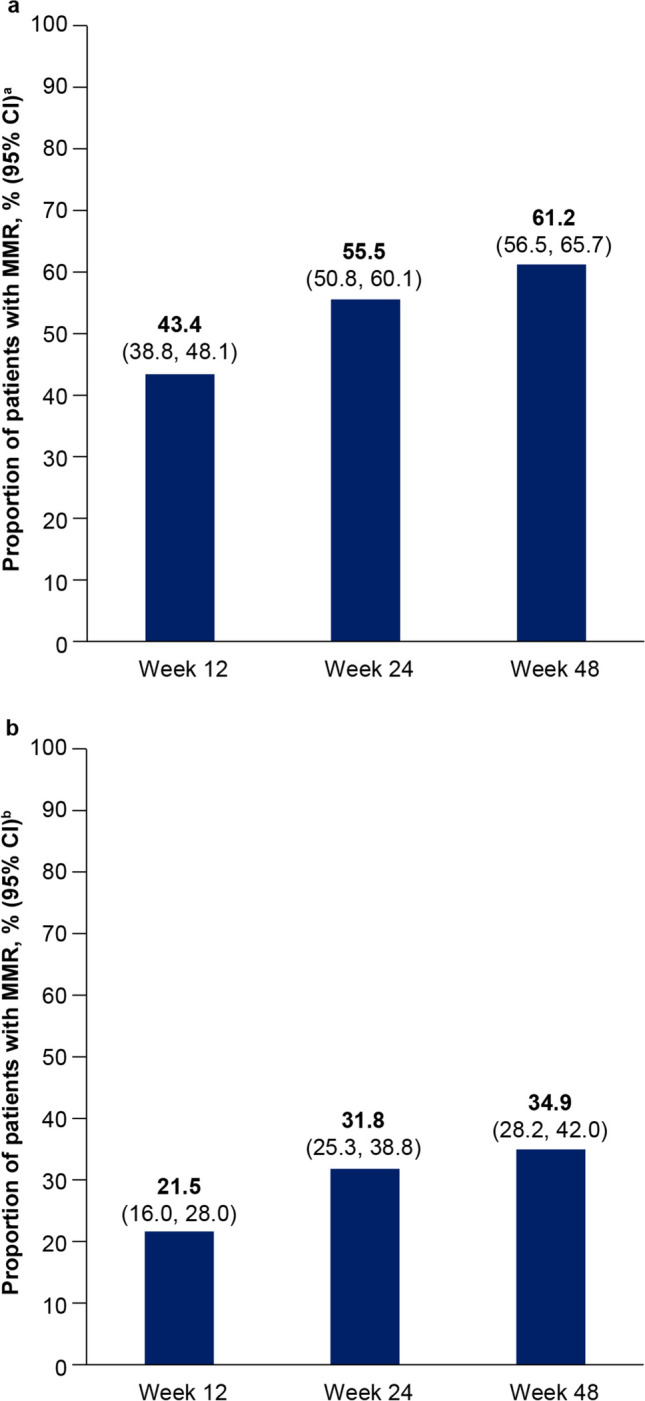
MMR rates by visit in **a** the MR analysis population, and **b** patients who did not have MMR at baseline. *CI* confidence interval, *MR* molecular response, *MMR* major molecular response ; ^a^MR analysis set, overall population (n = 456); ^b^MR analysis set, MMR not achieved at baseline (n = 195); n used as denominator to calculate proportion (%) of patients for each category. For the final assessment, the last assessment after the start of treatment with asciminib was used. Results of the last assessment were 61.2% (95% CI 56.5, 65.7) for the MR analysis set (overall) and 34.9% (95% CI 28.2, 42.0) for patients in the MR analysis set who had not achieved MMR at baseline. For the cumulative MMR, only assessment results obtained after the start of asciminib treatment were included. The Clopper-Pearson method was used to calculate 95% CIs

In the molecular response (MR) analysis set (n = 456), at the time that asciminib was initiated, 180 patients (39.5%, 95% CI 35.0, 44.1) had already achieved MMR, whereas 195 patients (42.8%) were not in MMR at baseline, and MR was not recorded or was not assessed in 81 patients [17.8%]. MMR rates (95% CI) were 43.4% (38.8, 48.1), 39.9% (35.4, 44.6), 32.2% (28.0, 36.7), and 59.6% (55.0, 64.2) at weeks 12, 24, 48 and at final assessment, respectively, in the MR analysis set. Cumulatively, the MMR rate by week 12 was 43.4%, rising steadily to 61.2% by week 48 of asciminib treatment (Fig. 3a). Similarly, for the 195 patients who had not achieved MMR at baseline, MMR rates (95% CI) were 21.5% (16.0, 28.0), 24.1% (18.3, 30.7), 17.9% (12.8, 24.1), and 32.8% (26.3, 39.9) at weeks 12, 24, 48 and at final assessment; the cumulative MMR rate rose from 21.5% by week 12 to 34.9% by week 48 (Fig. 3b) (Supplementary Table 7).

MMR rate at week 48 was investigated by patient characteristics and by previous treatment and treatment line (Table 6). There was no notable difference between the age categories (≥ 65 years vs. < 65 years) for cumulative MMR rate by week 48. For patients with concurrent renal impairment, hepatic impairment, or cardiac dysfunction, the cumulative MMR rate by week 48 was similar to that of patients without these conditions. When treatment line was considered, asciminib was effective as both a second-line and as a third-line or later-line treatment, with cumulative MMR rates of 85.7% and 60.4%, respectively, by week 48. Of the treatment options available, the most common previous therapies for patients were 1G and 2G TKIs, and the cumulative MMR rate by week 48 was 65.8% in patients who had received both previously and 74.4% in patients who had received 2G TKIs only.

**Table 6:** MMR achieved by week 48 by patient characteristics (molecular response analysis set)

Molecular response analysis set n = 456	m	MMR achievement (by week 48, cumulative)	
Factors		n (%)	(95% CI)
Sex			
Male	275	171 (62.2)	(56.2, 67.9)
Female	181	108 (59.7)	(52.1, 66.9)
Age (elderly)			
< 65 years	190	120 (63.2)	(55.9, 70.0)
≥ 65 years	266	159 (59.8)	(53.6, 65.7)
Duration of CML			
< 1 year	62	39 (62.9)	(49.7, 74.8)
≥ 1 year to < 5 years	221	129 (58.4)	(51.6, 64.9)
≥ 5 years	169	108 (63.9)	(56.2, 71.1)
Concurrent condition			
None	128	69 (53.9)	(44.9, 62.8)
Yes	328	210 (64.0)	(58.6, 69.2)
Concurrent condition: renal impairment			
None	400	241 (60.3)	(55.3, 65.1)
Yes	56	38 (67.9)	(54.0, 79.7)
Concurrent condition: hepatic impairment			
None	441	269 (61.0)	(56.3, 65.6)
Yes	15	10 (66.7)	(38.4, 88.2)
Concurrent condition: cardiac impairment			
None	364	222 (61.0)	(55.8, 66.0)
Yes	92	57 (62.0)	(51.2, 71.9)
Medical history^a^			
None	247	152 (61.5)	(55.2, 67.6)
Yes	209	127 (60.8)	(53.8, 67.4)
Treatment line number of asciminib			
1	0	–	(NE, NE)
2	14	12 (85.7)	(57.2, 98.2)
≥ 3	442	267 (60.4)	(55.7, 65.0)
Type of previous treatment			
1G TKI	2	2 (100)	(15.8, 100.0)
2G TKI	133	99 (74.4)	(66.2, 81.6)
3G TKI	0	–	(NE, NE)
1G + 2G TKI	155	102 (65.8)	(57.8, 73.2)
2G + 3G TKI	99	45 (45.5)	(35.4, 55.8)
1G + 3G TKI	2	1 (50.0)	(1.3, 98.7)
1G + 2G + 3G TKI	65	30 (46.2)	(33.7, 59.0)
Presence/absence of resistance/intolerance to previous drugs			
Resistance	62	22 (35.5)	(23.7, 48.7)
Intolerance	243	173 (71.2)	(65.1, 76.8)
Resistance and intolerance	151	84 (55.6)	(47.3, 63.7)
Others	0	–	(NE, NE)
*BCR::ABL1* mutation			
Unmeasured	317	220 (69.4)	(64.0, 74.4)
Negative	118	51 (43.2)	(34.1, 52.7)
Positive	21	8 (38.1)	(18.1, 61.6)

m represents the number of patients in the applicable category and n represents the cumulative number of patients achieving MMR (the denominator for the proportion is m)

For MMR achievement (by week 48, cumulative), only assessments after the start of asciminib treatment were cumulated

The Clopper-Pearson method was used to calculate 95% CIs

*CI* confidence interval, *CML* chronic myeloid leukemia, *G* generation, *NE* not estimable, *MMR* major molecular response, *TKI* tyrosine kinase inhibitor

^a^Significant medical history that had resolved prior to start of asciminib treatment

#### Deep molecular, cytogenetic, and hematological responses

**Fig. 4:**
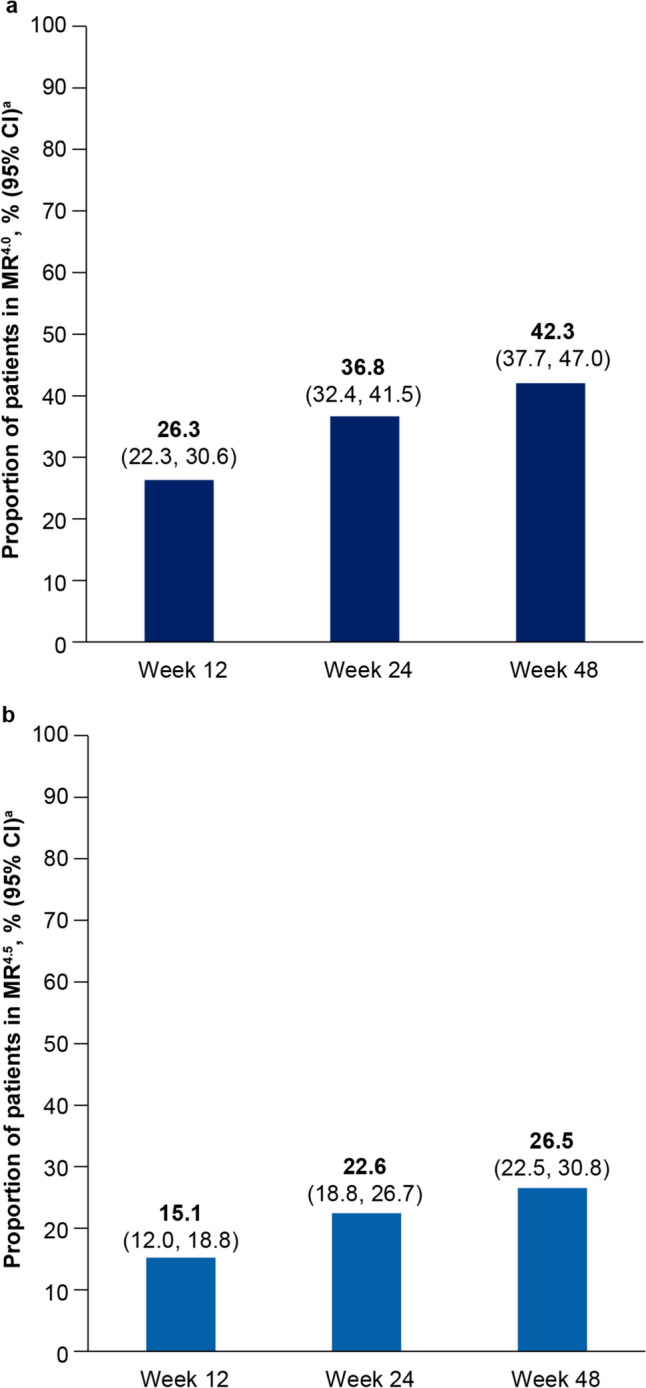
Rates by visit for **a** MR^4.0^ and **b** MR^4.5^ in the MR analysis population. *CI* confidence interval, *MR* molecular response, *MMR* major molecular response ; ^a^MR analysis set, overall population (n = 456) used as denominator to calculate proportion (%) of patients for each category. For the final assessment, the last assessment after the start of treatment with asciminib was used. For the cumulative rates, only assessment results after the start of asciminib treatment were included. The Clopper–Pearson method was used to calculate 95% CIs

Deep molecular responses (MR^4.0^ and MR^4.5^) were also achieved by patients in the MR analysis set. At baseline, the MR^4.0^ rate (95% CI) was 21.3% (17.6, 25.3) and at week 48 of asciminib treatment it was 22.6% (18.8, 26.7). The cumulative MR^4.0^ rate was 42.3% by week 48 (Fig. 4a). Similarly, the MR^4.5^ rate (95% CI) was 13.2% (10.2, 16.6) at baseline and 14.0% (11.0, 17.6) at week 48. By week 48, the cumulative rate was 26.5% (Fig. 4b). For patients who had not achieved MR^4.0^ or MR^4.5^ before starting asciminib (n = 278 and 315, respectively), by week 48 the cumulative MR^4.0^ and MR^4.5^ rates (95% CI) were 24.5% (19.5, 30.0) and 16.5% (12.6, 21.1), respectively. CCyR was reported in 48.8% of patients at baseline and by week 48 the cumulative rate was 53.6%; likewise, 74.7% of patients had a CHR at baseline with a cumulative rate of 85.2% by week 48.

#### Effectiveness in patients with *BCR::ABL1* mutations at baseline

At baseline, 25 of the 523 patients in the safety analysis set had *BCR::ABL1* mutations. Supplementary Table 8 shows the responses achieved in these patients.

During the observation period, *BCR::ABL1* mutations were identified in 5 patients (0.9%). A 69-year-old male and an 89-year-old female both developed a T315I mutation after 78 days (molecular response was > 1% at week 12) and 19 days (molecular response not measured) of treatment, respectively. A 53-year-old male developed a T315L mutation after 31 days of treatment (molecular response was > 1% at week 12) and a 63-year-old male developed p.C475fs*11 and c.1423_1424ins mutations after 193 days of treatment (molecular response was ≤ 1% week 24). In a 29-year-old female, A337T and P465S mutations were detected after 202 days of treatment; this patient progressed from a molecular response of ≤ 0.0032% to ≤ 0.1% at week 24, with a final assessment of ≤ 1% at week 24. All 5 patients discontinued treatment.

## Discussion

The phase 3 ASCEMBL study had previously demonstrated the efficacy and safety of asciminib in patients with CML-CP who had received ≥ 2 TKIs, including in a subgroup of Japanese patients [8, 9]. To further investigate asciminib in a more diverse Japanese patient population in clinical practice, the current PMS study was initiated. Notably, all patients in Japan who had received asciminib were eligible, including those patients who would be ineligible for clinical studies; therefore, the outcomes should be representative of a real-world population and provide a fair reflection of the effectiveness and safety of asciminib in Japanese patients.

In Japan, asciminib is now approved for patients with CML at any treatment stage [18, 19]; however, for the duration of this PMS study, asciminib was approved for the treatment of resistant or intolerant CML-CP. Therefore, as expected, patients who participated in this PMS study had CML of long duration and were heavily pretreated, most commonly with 2G TKIs. In ASCEMBL, intolerance was a greater influencer of discontinuation of previous TKIs than treatment failure [8] and in the ASC4OPT trial, 28.4% of patients had discontinued previous TKIs due to intolerance before switching to asciminib [29]. Similarly, intolerance was the main reason for switching from TKI to asciminib in this PMS study.

In Japan, 80 mg qd is the current approved regimen for asciminib [18], but at the time of this PMS study it was 40 mg bid; both regimens have been shown to have similar exposure, high efficacy and good tolerability [29, 30]. Notably, over half of patients in this PMS study initiated asciminib at the approved daily dose of 80 mg (40 mg bid), however, a third initiated treatment at the lower dose of 40 mg daily. The lower dose is indicated based on the condition of individual patients [18] and its common use is, therefore, a reflection of clinical practice in Japan. Similarly, in real-world studies conducted outside Japan, patients generally received asciminib at the approved dose (40 mg bid, or as either 80 mg qd or 40 mg bid in the US) with lower doses used in some patients, but higher doses were used for patients with T315I mutation or advanced disease [31–34] indicating that appropriate adjustments were made for individuals. In this PMS study, the approved dose (40 mg bid) remained the most frequently used dose and was not exceeded, importantly, patients remained on asciminib for a relatively long period, which suggests that asciminib dosing was optimal for individuals from the start of treatment and was sufficiently well tolerated and effective that adjustments were not required in most patients. This stability in the asciminib dose over time in clinical practice is likely to have a positive impact on patient outcomes, as demonstrated by the durable efficacy and safety reported for ASCEMBL [10, 11] even after 4 years of follow-up [12].

Five of the 6 pre-defined safety specifications were observed in this PMS study, photosensitivity was not observed. Treatment discontinuations due to ADR of the safety specifications were low and although not directly comparable, the pattern and incidences of safety specifications resulting in discontinuation reflect those for these same AEs leading to discontinuation in ASCEMBL [35]. These observations suggest that these events with asciminib were tolerated or managed, thus avoiding the need to discontinue or switch treatment. No new safety signals were observed for these safety specifications, which supports the overall observation for safety in ASCEMBL [8, 9].

Asciminib was generally well tolerated during this PMS study. The majority of ADRs were low-grade events (grade 1 or 2) and no ADRs of particularly high incidence nor ADRs of new interest were observed, which reflects the predominantly lower-grade treatment-related AEs and overall findings in ASCEMBL [8, 9]. Notably, however, the incidence rate of ADRs observed during this PMS (33.1%) was lower than the treatment-related events for asciminib observed in ASCEMBL (64.0%) and in the ASCEMBL Japanese subgroup (46.2%) [8, 9]. In ASC4OPT and ASCEMBL, the discontinuation rates due to AEs were low (6.0% and 5.8%, respectively) [8, 29], which although not directly comparable is aligned with the low rates of ADRs resulting in discontinuations reported during this PMS.

Age is an important factor when choosing appropriate CML treatments due to age-related complexities, such as comorbidities [3]. However, although a numerically higher ADR incidence rate was observed in patients aged ≥ 65 years versus those < 65 years in this study, no new safety concerns were identified in this patient group. Similarly, no notable trends in the incidence or types of ADR were observed in patients with renal or hepatic impairment or cardiac dysfunctioncompared with patients without them. Previously treated patients and those with comorbidities might be expected to experience more ADRs due to the complexities of their condition and continued treatment. Numerically higher rates of ADRs were noted with increasing lines of treatment during this PMS; however, similar rates of discontinuation were observed, which suggests that asciminib is tolerated by patients regardless of its place in the treatment sequence.

The MR analysis set included all patients regardless of whether response had been achieved previously or not. In this mixed population, the cumulative MMR rate increased steadily, including for those patients who were not in MMR at baseline; for these patients, rates reached 34.9% by week 48. Similar rates were reported in ASCEMBL, which was conducted in patients who had not achieved MMR: weeks 48 and 96 cumulative MMR were 35.0% and 42.7%, respectively [10]. Thus, the results of this PMS study are consistent with those of ASCEMBL [8, 10], but interestingly, asciminib also provided clinical benefit to patients who had already achieved some level of response with previous TKIs in this PMS. This is consistent with outcomes from ASC4OPT, where most patients already in MMR at the start of asciminib treatment maintained their MMR status at week 48 of asciminib treatment [29]. Importantly, the MMR rates in this PMS study were not influenced by patient characteristics or the presence of comorbidities, which suggests that patients receiving asciminib have the potential to achieve clinical benefit regardless of previous therapies, patient characteristics, or concurrent disease. Additionally, MR^4.0^ and MR^4.5^ were achieved or maintained in a substantial proportion of patients, showing the depth of response achieved with asciminib in this broad patient population and confirming observations in ASCEMBL [10, 11]. Asciminib’s effectiveness was also confirmed in some patients who had *BCR::ABL1* mutations at baseline, including those with V299L, T315I, E255K, and G250E mutations. This is notable as the ASCEMBL and ASC4OPT studies excluded patients with T315I or V299L at baseline [8, 29]; however, patients with other *BCR::ABL1* mutations responded to asciminib and were in MMR at week 24 [8]. In this PMS study, genetic mutations observed post baseline resulted in treatment discontinuation.

As an observational study, this PMS did not include a control group for comparison and did not analyze outcomes in patients who were not exposed to asciminib. Therefore, it is not possible to definitively assign the findings to asciminib exposure. In addition, we acknowledge that there was a relatively high rate of patients whose MMR assessment was not recorded/not measured, which may have affected the accurate assessment of MMR at individual timepoints; however, substantial asciminib effectiveness was observed despite the exclusion of these patients. In addition, effectiveness was based on physician evaluation rather than central review and, therefore, may have been less consistent across clinics and physicians.

Treatment options are limited for patients who have received multiple TKIs and discontinued treatment due to intolerance; therefore, treatments with favorable safety and tolerability profiles that can maintain effectiveness and remain tolerable are important. Collectively, these PMS data show that asciminib was sufficiently well tolerated with no new safety concerns and low rates of discontinuation, and that patients were able to remain on treatment and achieve or maintain response, including patients with comorbidities. Ultimately, this may help increase the proportion of patients eligible for TFR and further contribute to their QOL.

## Supplementary Information

Below is the link to the electronic supplementary material.

Supplementary file1 (PDF 2381 KB)

## Data Availability

Novartis is committed to sharing access to patient-level data and supporting clinical documents from eligible studies with qualified external researchers. These requests are reviewed and approved by an independent review panel based on scientific merit. All data provided are anonymized to respect the privacy of patients who have participated in the study in line with applicable laws and regulations. This study data availability is according to the criteria and process described on www.clinicalstudydatarequest.com.
