# Supplementary material for: Postmarketing surveillance study of asciminib in patients with resistant/intolerant chronic myeloid leukemia in Japan
Source: Int J Hematol. 2026 Mar 31;124(2):177–91. doi: 10.1007/s12185-026-04199-x (PMC13407570; doi:10.1007/s12185-026-04199-x)
Supplement: Supplementary file 1 — Supplementary file1 (PDF 2381 KB) [file 12185_2026_4199_MOESM1_ESM.pdf]

Postmarketing surveillance study of asciminib in patients with resistant/intolerant chronic myeloid leukemia in Japan

Kazuaki Yamaguchi, Makoto Aoki, and Ryohei Osako

Contents

1. Supplementary Information 3

1.1 Study population sample size calculation 3

1.2 Analysis sets 3

1.3 Assessments 5

1.4 Factorial analysis of safety specifications 6

Supplementary Table 1 Safety specifications and definitions 7

Supplementary Table 2 Previous drugs used before the initiation of asciminib (safety analysis set) 10

Supplementary Table 3 Dose adjustments and compliance to treatment (safety analysis set) 11

Supplementary Table 4 Incidence of ADRs leading to treatment interruption by grade (safety analysis set) 13

Supplementary Table 5 Incidence of the most common ADRs, serious ADRs, and ADRs leading to discontinuation and interruption (all grade and grade ≥3) by safety specification (safety analysis set) 14

Supplementary Table 6 Incidence of ADR by asciminib treatment line (safety analysis set) 17

Supplementary Table 7 MMR status at each timepoint and cumulatively in patients by the MMR status at the start of asciminib treatment (molecular response analysis set) 18

Supplementary Table 8 Effectiveness in patients who had *BCR::ABL1* mutations at baseline 19

References 22

1. Supplementary Information

1.1 Study population sample size calculation

Rationale: The sample size was chosen to understand the safety profile of asciminib after approval, including the incidences of the safety specifications (myelosuppression, infections, QT interval prolongation, pancreatitis, vascular occlusive events, and photosensitivity), actions taken for asciminib and exploration of patient characteristics and other factors possibly affecting the occurrence of these adverse events (AEs).

Assuming that the rates of AEs for the safety specifications observed for asciminib in the phase 3 ASCEMBL study [1] would be similar in this postmarketing surveillance (PMS) study, the number of patients needed to maintain the width of the 95% confidence intervals (CIs) of these rates within 10% were the following:

- 379 patients for myelosuppression
- 396 patients for infections
- 79 patients for QT interval prolongation
- 135 patients for pancreatitis
- 104 patients for vascular occlusive events
- 35 patients for photosensitivity

Therefore, a target sample size of at least 400 patients (as safety analysis set) was determined to allow a reasonable assessment of the safety specifications of asciminib.

1.2 Analysis sets

The safety analysis set included case report form (CRF)-locked patients (all patients whose CRFs were locked), excluding those who met any of the following criteria:

- Observation not within the contract term. Patients in whom the safety observation was performed after the end date of the contract term including the follow-up period

- Administration not within the registration period. Patients whose start date of treatment with asciminib was not within the protocol-specified study registration period
- No investigator's signature and no handwriting name with the seal
- Registered but not within the registration period. Patients whose registration date was not within the protocol-specified study registration period
- Registered but registration time limit was not observed. Patients whose start date of treatment in the CRF data on treatment with asciminib and registration date, when compared, showed a deviation from the protocol-specified registration period
- Previous use of asciminib
- Off-label use/disease not targeted by this study
- Protocol criteria deviations. Patients who did not meet the inclusion criteria
- Multiple registration. Patients who were found to be one and the same person through notification by the site and whose registration date was later than the oldest date
- Patient not registered. Patients not registered but whose CRFs were collected
- Registration not confirmed. Patients whose registration had not been confirmed
- Asciminib not administered
- Start date of asciminib unknown/not recorded
- Adverse event occurrence unknown/not recorded
- Other patients (who were assessed as ones who should be excluded from safety analyses for other reasons)

Molecular response, cytogenetic response, and hematological response analysis sets included those patients from the safety analysis set excluding those who met any of the following criteria**:**

- Molecular response/cytogenetic response/ hematological response assessment missing/not recorded

  Patients in whom molecular response/cytogenetic response/hematological response was not assessed or assessments were not recorded both before and after treatment with asciminib

- Others (patients who were excluded from the effectiveness analysis set for other reasons)

1.3 Assessments

Patient characteristics and outcomes from laboratory tests were collected at the start of treatment, including the following:

- Sex, age, reasons for asciminib use, date of diagnosis, medical history, concurrent conditions, previous drugs for underlying disease, presence/absence of resistance/intolerance to previous drugs and details, treatment line number, *BCR::ABL1* mutations

During the observation period (weeks 12, 24, 48 and at discontinuation), assessments included the following:

- Asciminib treatment, treatment discontinuation/interruptions, concomitant medications and therapies
- Laboratory tests for vital signs (blood pressure, electrocardiogram [ECG, QTc interval]), hematology (white blood cell [WBC] count, differential WBC count [neutrophil, lymphocyte, basophil, eosinophil, monocyte], platelet count) and blood chemistry (amylase, lipase)
- Treatment response was assessed according to European LeukemiaNet (ELN) 2020 recommendations [2] and JSH Practical Guidelines for Hematological Malignancies [3] and were recorded before the start of treatment (within 30 days before the start date of treatment) and during the observation period (weeks: 12, 24, 48 and at discontinuation)
    - MMR ($BCR::ABL1^{IS}$ ≤0.1%), $MR^{4.0}$ ($BCR::ABL1^{IS}$ ≤0.01%), and $MR^{4.5}$ ($BCR::ABL1^{IS}$ ≤0.0032%)
    - Complete cytogenetic response (CCyR, in patients with bone marrow aspiration only; Philadelphia chromosome-positive [Ph+] cell=0%)
    - Complete hematologic response (CHR, meeting all six criteria: WBC count <10,000/µL; platelet count <450,000/µL; no blast cell and promyelocyte in

peripheral blood; myelocyte + metamyelocyte in peripheral blood = 0%; basophil <5%; no spleen and liver swelling, and no extramedullary lesion)

- Presence of *BCR::ABL1* mutations before and during the observation period.
- Presence/absence of AEs, AE term, date of occurrence, seriousness, severity (Common Terminology Criteria for Adverse Events [CTCAE] grade 1–5), causal relationship to asciminib, actions taken for asciminib to manage AEs, suspected non-asciminib factors, outcome, date of outcome, and laboratory test values related to AEs.

1.4 Factorial analysis of safety specifications

To investigate patient factors that may affect safety, the incidence rates of adverse drug reactions (ADRs) were analyzed by subgroup for the following patient factors and the odds ratios and 95% confidence intervals (CIs) calculated:

- Sex, age, duration of CML, concurrent condition, medical history, smoking history, number of smoking years, previous drugs for underlying disease, treatment line number of asciminib, type of previous treatment, presence/absence of resistance/intolerance to previous drugs, *BCR::ABL1* gene mutation, pregnancy during the observational period

The patient factors with subgroups having odds ratios with 95% CI excluding 1 were considered for investigation as risk factors. Examining the incidence of ADRs, the subgroup "age (elderly) ≥65 years" had 95% CI of the odds ratio that did not include 1, suggesting that it may contribute to the high incidence rate of ADRs. Of note, the lower limit of the 95% CI of the odds ratio for "age (elderly): ≥65 years" is 1.00 due to rounding (calculated value was slightly higher than 1)

Supplementary Table 1 Safety specifications and definitions

Safety specifications	Definition
**Important identified risks**	
Pancreatitis	Abdominal distension (PT)
	Abdominal pain (PT)
	Abdominal pain upper (PT)
	Abdominal rigidity (PT)
	Abdominal tenderness (PT)
	Acute abdomen (PT)
	Amylase increased (PT)
	Ascites (PT)
	Blood bilirubin increased (PT)
	Gastrointestinal pain (PT)
	Hyperbilirubinemia (PT)
	Ileus paralytic (PT)
	Jaundice (PT)
	Lipase increased (PT)
	Lipase urine increased (PT)
	Nausea (PT)
	Pancreatic enzyme abnormality (PT)
	Pancreatic hemorrhage (PT)
	Pancreatic pseudocyst (PT)
	Pancreatic pseudocyst drainage (PT)
	Pancreatitis (PT)
	Pancreatitis acute (PT)
	Pancreatitis hemorrhagic (PT)
	Pancreatitis necrotizing (PT)
	Pancreatitis relapsing (PT)
	Vomiting (PT)
	Vomiting projectile (PT)
	Pancreatic abscess (PT)
	Peripancreatic fluid collection (PT)
	Edematous pancreatitis (PT)
	Abdominal rebound tenderness (PT)
	Lipase abnormal (PT)
	Pancreatorenal syndrome (PT)
	Pancreatic phlegmon (PT)

Safety specifications	Definition
	Abdominal compartment syndrome (PT)
	Cullen's sign (PT)
	Hemorrhagic ascites (PT)
	Fat necrosis (PT)
	Pancreatic enzymes abnormal (PT)
	Pancreatic enzymes increased (PT)
	Hyperamylasemia (PT)
	Blood trypsin increased (PT)
	Ischemic pancreatitis (PT)
	Gastrointestinal sounds abnormal (PT)
	Bilirubin conjugated abnormal (PT)
	Hyperlipasemia (PT)
	Intestinal atony (PT)
	Intra-abdominal pressure increased (PT)
	Amylase abnormal (PT)
	Amylase creatinine clearance ratio abnormal (PT)
	Grey Turner's sign (PT)
	Hemorrhagic necrotic pancreatitis (PT)
	Pancreatic duct rupture (PT)
	Ultrasound pancreas abnormal (PT)
	Pancreatic pseudoaneurysm (PT)
	Pancreatic cyst drainage (PT)
	Abdominal wall edema (PT)
	Computerized tomogram pancreas abnormal (PT)
	Immune-mediated pancreatitis (PT)
	Pancreatic pseudocyst rupture (PT)
	Pancreatic pseudocyst hemorrhage (PT)
	Subacute pancreatitis (PT)
	Walled-off pancreatic necrosis (PT)
	Bile acids increased (PT)
	Idiopathic pancreatitis (PT)
	Bile acids abnormal (PT)
	Cholecystokinin increased (PT)
	Hemorrhagic pancreatic cyst (PT)
Myelosuppression	Hematopoietic thrombocytopenia (SMQ)[a]
	Hematopoietic leukopenia (SMQ)[a]
	Hematopoietic erythropenia (SMQ)

Safety specifications	Definition
	Hematopoietic cytopenias affecting more than one type of blood cell (SMQ)[a]
QT interval prolongation	Torsade de pointes/QT prolongation (SMQ)
Vascular occlusive events	Ischemic central nervous system vascular conditions (SMQ)
	Embolic and thrombotic events, arterial (SMQ)
	Ischemic heart disease (SMQ)[a]
Infections	Infections and infestations (SOC)
**Important potential risk**	
Photosensitivity	Juvenile spring eruption (PT)
	Photoonycholysis (PT)
	Photosensitivity reaction (PT)
	Polymorphic light eruption (PT)
	Pseudoporphyria (PT)
	Solar dermatitis (PT)
	Solar urticaria (PT)
	Sunburn (PT)
	Photodermatosis (PT)
	Injection site photosensitivity reaction (PT)
	Application site photosensitivity reaction (PT)
	Infusion site photosensitivity reaction (PT)
	Retinal phototoxicity (PT)
	Chronic actinic dermatitis (PT)
	Implant site photosensitivity (PT)
	Hydroa vacciniforme (PT)

MedDRA/J, Japanese version of Medical Dictionary for Regulatory Activities; PT, preferred term; SMQ, standardized MedDRA queries. [a] SMQ (Narrow).
MedDRA/J version 27.0.

Supplementary Table 2 Previous drugs used before the initiation of asciminib (safety analysis set)

Safety analysis set (n = 523)		Reason for switching drugs			
		Resistance	Intolerance	Resistance and intolerance	Others
Drug name	m (%)[a]	n (%)	n (%)	n (%)	n (%)
Imatinib	256 (48.9)	99 (38.7)	148 (57.8)	3 (1.2)	6 (2.3)
Dasatinib	426 (81.5)	113 (26.5)	305 (71.6)	6 (1.4)	2 (0.5)
Nilotinib	278 (53.2)	85 (30.6)	181 (65.1)	2 (0.7)	10 (3.6)
Bosutinib	329 (62.9)	65 (19.8)	259 (78.7)	2 (0.6)	3 (0.9)
Ponatinib	189 (36.1)	78 (41.3)	106 (56.1)	1 (0.5)	4 (2.1)
Others	0	-	-	-	-

TKI, tyrosine kinase inhibitor. [a] m represents the number of patients with a history of TKI use.

Safety analysis set (n = 523) was used as the denominator to calculate the proportions (%) of patients with history of TKI use, whereas m was the denominator for the proportion (%) of reasons for switching drugs. When the same patient received the same drug multiple times, the patient was counted once in "m." When the same reason for switching drugs occurred multiple times for the same patient and for the same drug, the corresponding reason for switching was counted once in "n." When a patient lacked data on "resistance" or "intolerance" and only had data on "others," the patient was categorized as "others."

Supplementary Table 3 Dose adjustments and compliance to treatment (safety analysis set)

	Safety analysis set (n = 523)
Asciminib treatment duration (days)[a]	
Mean (standard deviation)	271.7 (112.31)
Median	336.0
Q1-Q3	217.0-336.0
Min-Max	1-336
Categories of treatment duration, n (%)	
<12 weeks	70 (13.4)
≥12 weeks to <24 weeks	42 (8.0)
≥24 weeks to <48 weeks	37 (7.1)
≥48 weeks	374 (71.5)
Categories of initial daily dose, n (%)	
<40 mg	29 (5.5)
40 mg	192 (36.7)
>40 mg to <80 mg	2 (0.4)
80 mg	300 (57.4)
>80 mg	0
Cumulative dose (mg)	
Mean (standard deviation)	17183.8 (9802.84)
Median	18680.0
Q1-Q3	7840.0-26880.0
Min-Max	80-26880
Mean daily dose (mg/day)	
Mean (standard deviation)	63.8 (20.76)
Median	79.2
Q1-Q3	40.0-80.0
Min-Max	2-80
Categories of mean daily dose, n (%)	
<40 mg/day	54 (10.3)
40 mg/day	94 (18.0)
>40 mg/day to <80 mg/day	117 (22.4)
80 mg/day	258 (49.3)
>80 mg/day	0

	Safety analysis set (n = 523)
Dose intensity (mg/day)	
Mean (standard deviation)	62.0 (22.02)
Median	76.9
Q1-Q3	40.0-80.0
Min-Max	2-80
Dose intensity category, n (%)	
<40 mg/day	77 (14.7)
40 mg/day	84 (16.1)
>40 mg/day to <80 mg/day	119 (22.8)
80 mg/day	243 (46.5)
>80 mg/day	0
Categories of the most frequent daily dose, n (%)	
<40 mg	43 (8.2)
40 mg	143 (27.3)
>40 mg to <80 mg	3 (0.6)
80 mg	334 (63.9)
>80 mg	0

Q, quartile. [a] Treatment duration: date of the last treatment - date of the first treatment + 1.
The number of patients for the safety analysis set (n = 523) was used as the denominator to calculate the proportions (%).

Supplementary Table 4 Incidence of ADRs leading to treatment interruption by grade (safety analysis set)

	Safety analysis set (n = 523)	
ADR (PT)[a]	All grade n (%)	Grade ≥3 n (%)
ADR Total	35 (6.7)	22 (4.2)
Platelet count decreased	9 (1.7)	6 (1.1)
Neutropenia	6 (1.1)	6 (1.1)
Thrombocytopenia	3 (0.6)	3 (0.6)
Pneumonia	2 (0.4)	2 (0.4)
Electrocardiogram QT prolonged	2 (0.4)	1 (0.2)
Pleural effusion	2 (0.4)	1 (0.2)
Amylase increased	2 (0.4)	0
Atrioventricular block complete	1 (0.2)	1 (0.2)
Cardiac failure	1 (0.2)	1 (0.2)
Cardiomyopathy	1 (0.2)	1 (0.2)
Hepatic function abnormal	1 (0.2)	1 (0.2)
Nephrogenic anemia	1 (0.2)	1 (0.2)
White blood cell count decreased	1 (0.2)	1 (0.2)
Bacteremia	1 (0.2)	0
Blood creatinine increased	1 (0.2)	0
Bronchopulmonary aspergillosis	1 (0.2)	0
Decreased appetite	1 (0.2)	0
Gastroenteritis salmonella	1 (0.2)	0
Herpes zoster	1 (0.2)	0
KL-6 increased	1 (0.2)	0
Lipase increased	1 (0.2)	0
Malaise	1 (0.2)	0
Myelosuppression	1 (0.2)	0
Rash	1 (0.2)	0

ADR, adverse drug reaction; KL-6, Krebs von den Lungen-6 glycoprotein; PT, preferred term. [a] ADRs were ranked from the highest to lowest incidence (all-grade events) then alphabetically for ADR with the same incidence.

The number of patients for the safety analysis set (n = 523) was used as the denominator to calculate the proportions (%).

Supplementary Table 5 Incidence of the most common ADRs, serious ADRs, and ADRs leading to discontinuation and interruption (all grade and grade ≥3) by safety specification (safety analysis set)

**Safety specification**<br>ADR category<br>Most common ADR	Safety analysis set (n = 523)<br>All grade<br>n (%)	 Grade ≥3<br>n (%)
**Myelosuppression**		
All ADRs[a]	45 (8.6)	32 (6.1)
Platelet count decreased	27 (5.2)	21 (4.0)
Neutropenia	14 (2.7)	12 (2.3)
Thrombocytopenia	7 (1.3)	5 (1.0)
Serious ADRs[b]	13 (2.5)	12 (2.3)
Platelet count decreased	5 (1.0)	5 (1.0)
Neutropenia	4 (0.8)	4 (0.8)
Anemia	2 (0.4)	2 (0.4)
Thrombocytopenia	1 (0.2)	1 (0.2)
ADRs leading to discontinuation	9 (1.7)	7 (1.3)
Platelet count decreased	5 (1.0)	5 (1.0)
Thrombocytopenia	2 (0.4)	1 (0.2)
Anemia	1 (0.2)	1 (0.2)
Neutropenia	1 (0.2)	1 (0.2)
Cytopenia	1 (0.2)	0
ADRs leading to interruption	17 (3.3)	13 (2.5)
Platelet count decreased	9 (1.7)	6 (1.1)
Neutropenia	6 (1.1)	6 (1.1)
Thrombocytopenia	3 (0.6)	3 (0.6)
Myelosuppression	1 (0.2)	0
WBC count decreased	1 (0.2)	1 (0.2)
**Infections**		
All ADRs[a]	7 (1.3)	3 (0.6)
Pneumonia	3 (0.6)	3 (0.6)
Herpes zoster	2 (0.4)	0
Serious ADRs[c]	4 (0.8)	3 (0.6)
Pneumonia	3 (0.6)	3 (0.6)
Bacteremia	1 (0.2)	0
Gastroenteritis salmonella	1 (0.2)	0

Safety specification<br>ADR category<br>Most common ADR	Safety analysis set (n = 523)<br>All grade<br>n (%)	 Grade ≥3<br>n (%)
ADRs leading to discontinuation	0	0
ADRs leading to interruption	4 (0.8)	2 (0.4)
Pneumonia	2 (0.4)	2 (0.4)
Bacteremia	1 (0.2)	0
Bronchopulmonary aspergillosis	1 (0.2)	0
Gastroenteritis salmonella	1 (0.2)	0
Herpes zoster	1 (0.2)	0
**QT interval prolongation**		
All ADRs	13 (2.5)	7 (1.3)
Electrocardiogram QT prolonged	13 (2.5)	7 (1.3)
Serious ADRs[d]	1 (0.2)	1 (0.2)
Electrocardiogram QT prolonged	1 (0.2)	1 (0.2)
ADRs leading to discontinuation	5 (1.0)	4 (0.8)
Electrocardiogram QT prolonged	5 (1.0)	4 (0.8)
ADRs leading to interruption	2 (0.4)	1 (0.2)
Electrocardiogram QT prolonged	2 (0.4)	1 (0.2)
**Pancreatitis**		
All ADRs[a]	23 (4.4)	4 (0.8)
Lipase increased	5 (1.0)	1 (0.2)
Nausea	5 (1.0)	1 (0.2)
Pancreatitis	5 (1.0)	2 (0.4)
Amylase increased	4 (0.8)	0
Abdominal pain upper	3 (0.6)	0
Vomiting	3 (0.6)	0
Pancreatic enzyme increased	2 (0.4)	0
Serious ADRs[e]	4 (0.8)	2 (0.4)
Pancreatitis	2 (0.4)	2 (0.4)
Abdominal distension	1 (0.2)	0
Amylase increased	1 (0.2)	0
Lipase increased	1 (0.2)	0
Nausea	1 (0.2)	0
Vomiting	1 (0.2)	0

	Safety analysis set (n = 523)	
**Safety specification**	All grade	Grade ≥3
ADR category	n (%)	n (%)
Most common ADR		
ADRs leading to discontinuation	8 (1.5)	2 (0.4)
Nausea	3 (0.6)	1 (0.2)
Pancreatitis	3 (0.6)	1 (0.2)
Vomiting	2 (0.4)	0
Abdominal distension	1 (0.2)	0
Amylase increased	1 (0.2)	0
Lipase increased	1 (0.2)	0
ADRs leading to interruption	2 (0.4)	0
Amylase increased	2 (0.4)	0
Lipase increased	1 (0.2)	0
**Vascular occlusive event**		
All ADRs	1 (0.2)	0
Cerebellar infarction (nonserious)	1 (0.2)	0
Serious ADRs	0	0
ADRs leading to discontinuation	0	0
ADRs leading to interruption	0	0
**Photosensitivity**		
All ADRs	0	0

ADR, adverse drug reaction; WBC, white blood cell. [a] Most common ADRs of ≥1% incidence for myelosuppression, ≥2 patients (0.4%) for infections and pancreatitis, and other categories ≥1 patient (0.2%). [b] Of 18 events of myelosuppression in 13 patients, 12 were resolved, 2 were resolving, and 4 were not resolved by the end of the safety follow-up period. [c] Of 5 events in 4 patients, 4 were resolved and 1 was resolving by the end of the safety follow-up period. [d] The 1 event that occurred in 1 patient resolved by the end of the safety follow-up period; [e] Of the 8 events that occurred in these 4 patients, 2 were resolved and 3 were resolving by the end of the safety follow-up period, while the other 3 events (abdominal distension, nausea, and vomiting) occurred in the same patient who died. The number of patients for the safety analysis set (n = 523) was used as the denominator to calculate the proportions (%).

Supplementary Table 6 Incidence of ADR by asciminib treatment line (safety analysis set)

	Asciminib treatment line							
	≥3 (n = 507)		3 (n = 217)		4 (n = 143)		≥5 (n = 147)	
	All grade, n (%)	Grade ≥3, n (%)	All grade, n (%)	Grade ≥3, n (%)	All grade, n (%)	Grade ≥3, n (%)	All grade, n (%)	Grade ≥3, n (%)
ADRs	170 (33.5)	60 (11.8)	64 (29.5)	17 (7.8)	53 (37.1)	22 (15.4)	53 (36.1)	21 (14.3)
Serious ADRs	30 (5.9)	22 (4.3)	12 (5.5)	6 (2.8)	7 (4.9)	6 (4.2)	11 (7.5)	10 (6.8)
ADRs leading to treatment discontinuation	51 (10.1)	17 (3.4)	23 (10.6)	6 (2.8)	13 (9.1)	5 (3.5)	15 (10.2)	6 (4.1)
ADRs leading to treatment interruption	35 (6.9)	22 (4.3)	14 (6.5)	4 (1.8)	12 (8.4)	10 (7.0)	9 (6.1)	8 (5.4)

ADR, adverse drug reaction.

The number of patients for each treatment line category was used as the denominator to calculate the proportions (%) within that category.

Supplementary Table 7 MMR status at each timepoint and cumulatively in patients by the MMR status at the start of asciminib treatment (molecular response analysis set)

Molecular response analysis set (n = 456)[a]

	MMR achieved		MMR achieved (cumulation)	
Assessment time point	n (%)	(95% CI)	n (%)	(95% CI)
Week 12	198 (43.4)	(38.8, 48.1)	198 (43.4)	(38.8, 48.1)
Not recorded/assessment missing	120 (26.3)	--	--	--
Week 24	182 (39.9)	(35.4, 44.6)	253 (55.5)	(50.8, 60.1)
Not recorded/assessment missing	195 (42.8)	--	--	--
Week 48	147 (32.2)	(28.0, 36.7)	279 (61.2)	(56.5, 65.7)
Not recorded/assessment missing	266 (58.3)	--	--	--
At the final assessment	272 (59.6)	(55.0, 64.2)	279 (61.2)	(56.5, 65.7)

MMR not achieved (n = 195)[b]

	MMR achieved		MMR achieved (cumulation)	
Assessment time point	n (%)	(95% CI)	n (%)	(95% CI)
Week 12	42 (21.5)	(16.0, 28.0)	42 (21.5)	(16.0, 28.0)
Not recorded/assessment missing	42 (21.5)	--	--	--
Week 24	47 (24.1)	(18.3, 30.7)	62 (31.8)	(25.3, 38.8)
Not recorded/assessment missing	89 (45.6)	--	--	--
Week 48	35 (17.9)	(12.8, 24.1)	68 (34.9)	(28.2, 42.0)
Not recorded/assessment missing	123 (63.1)	--	--	--
At the final assessment	64 (32.8)	(26.3, 39.9)	68 (34.9)	(28.2, 42.0)

CI, confidence interval; MMR, major molecular response. [a] All patients included in molecular analysis set (regardless of attaining MMR at start of treatment); [b] Patients in the molecular analysis set who had not achieved MMR by the start of asciminib treatment. Respective analysis sets (n = 456 and 195) were used as the denominators to calculate the proportion (%) in the respective categories. At the final assessment, the last assessment after the start of asciminib treatment was considered. Patients who had discontinued patients and had no assessment after the day of discontinuation were summarized under "Not recorded/assessment missing". For MMR achieved (cumulation), only assessments after the start of asciminib treatment were cumulated. The Clopper-Pearson method was used to calculate 95% CIs.

Supplementary Table 8 Effectiveness in patients who had *BCR::ABL1* mutations at baseline

Age/sex	Mutation type in *BCR::ABL1*	Assessment point	Molecular response
25/male	T315I	At baseline	≤0.01% (MR$^{4.0}$ achieved)
		Week 12	≤0.0032% (MR$^{4.5}$ achieved)
		Week 24	≤0.0032% (MR$^{4.5}$ achieved)
		Week 48	≤0.0032% (MR$^{4.5}$ achieved)
79/male	Unknown	At baseline	≤0.01% (MR$^{4.0}$ achieved)
		Week 12	≤0.0032% (MR$^{4.5}$ achieved)
		Week 24	≤ 0.0032% (MR$^{4.5}$ achieved)
		Week 48	≤ 0.0032% (MR$^{4.5}$ achieved)
22/male	E255K	At baseline	Not measured
		Week 12	≤0.0032% (MR$^{4.5}$ achieved)
		Week 24	Not measured
81/male	T315I	At baseline	>1%
		Week 12	Not measured
		Week 24	Not measured
74/female	F317L, F359V	At baseline	≤0.1% (MMR achieved)
		Week 12	≤1%
34/male	V299L	-[a]	Not measured
		At baseline	≤0.1% (MMR achieved)
		Week 12	≤0.01% (MR$^{4.0}$ achieved)
		Week 24	≤0.01% (MR$^{4.0}$ achieved)
55/female	T315I	At baseline	>1%
		Week 12	>1%
86/female	Unknown	At baseline	≤0.01% (MR$^{4.0}$ achieved)
		Week 24	≤0.0032% (MR$^{4.5}$ achieved)
		Week 48	≤0.0032% (MR$^{4.5}$ achieved)
69/female	Unknown	-[a]	Not measured
		Week 24	≤0.0032% (MR$^{4.5}$ achieved)
		Week 48	Not measured
75/male	Unknown	At baseline	≤0.0032% (MR$^{4.5}$ achieved)
		Week 12	≤0.0032% (MR$^{4.5}$ achieved)
78/female	G250E, E255V	At baseline	≤1%

Age/sex	Mutation type in *BCR::ABL1*	Assessment point	Molecular response
		Week 12	≤0.0032% (MR$^{4.5}$ achieved)
77/male	c.550_822del, p.L184_K274del	At baseline	>1%
		Week 12	>1%
		Week 48	>1%
72/male	T315I	At baseline	>1%
		Week 12	>1%
		Week 24	>1%
		Week 24[a]	Not measured
67/male	p.L184_K274del, c.550_822del, p.R362fs*21, c.1086_1270del	At baseline	>1%
		Week 12	>1%
		Week 24	>1%
		Week 48	>1%
83/male	E255K	At baseline	>1%
		Week 12	>1%
		Week 24	>1%
		Week 48	>1%
80/female	T315I	At baseline	≤1%
		Week 12	>1%
		Week 24	Not measured
76/male	V299L	At baseline	>1%
		Week 12	>1%
		Week 12[a]	>1%
41/male	p.L184_K274del, c.550_822del		
77/female	T315I	At baseline	>1%
		Week 12	Not measured
65/female	F317L		
40/female	E255V, R307W, F317L		
48/male	T315I	At baseline	>1%
		Week 12	>1%
58/female	T315I, G250E		

Age/sex	Mutation type in *BCR::ABL1*	Assessment point	Molecular response
33/female	p.L184_K274del, c.550_822del	At baseline	>1%
72/female	I418V	At baseline	>1%
		Week 12	Not measured
		Week 24	>1%
		Week 48	>1%

MMR, major molecular response; MR, molecular response. [a] Data excluded from the analysis.
Dash denotes not applicable. Note that some patients did not have their molecular response measured within the predefined window and thus their molecular response is blank.

References

1. Pharmaceuticals and Medical Devices Agency. Review report. Scemblix Tablets. https://www.pmda.go.jp/drugs/2022/P20220330001/300242000_30400AMX00189_A100_1.pdf. Accessed November 10, 2025.

2. Hochhaus A, Baccarani M, Silver RT, Schiffer C, Apperley JF, Cervantes F, et al. European LeukemiaNet 2020 recommendations for treating chronic myeloid leukemia. Leukemia. 2020;34:966–84.

3. The Japanese Society of Hematology. Hematologic Tumor Treatment Guidelines. Practical Guidelines for Hematological Malignancies, 2018 (revised version 2020): Kanehara Publishing.
